# Biopolymer-based emulsions for the stabilization of *Trichoderma atrobrunneum* conidia for biological control

**DOI:** 10.1007/s00253-023-12381-y

**Published:** 2023-01-23

**Authors:** Yolanda Martínez, Markus Heeb, Tine Kalač, Zennat Gholam, Francis W.M.R. Schwarze, Gustav Nyström, Kevin De France

**Affiliations:** 1 Laboratory for Cellulose & Wood Materials, Empa – Swiss Federal Laboratories for Materials Science and Technology, Lerchenfeldstrasse 5, 9014 St. Gallen, Switzerland; 2 Laboratory for Cellulose & Wood Materials, Empa – Swiss Federal Laboratories for Materials Science and Technology, Überlandstrasse 129, 8600, Dübendorf, Switzerland; 3 Department of Health Science and Technology, ETH Zürich, Schmelzbergstrasse 9, 8092 Zürich, Switzerland; 4 Department of Chemical Engineering, Queen’s University, 19 Division St, Kingston, Ontario K7L 3N6 Canada

**Keywords:** *Trichoderma*, Emulsions, Agar, Cellulose nanocrystals, Biological control

## Abstract

**Abstract:**

*Trichoderma* spp. are ubiquitous soil-borne fungi that are widely used in biological control to promote and regulate healthy plant growth, as well as protect against plant pathogens. However, as with many biological materials, the relative instability of *Trichoderma* propagules limits its practical use in industrial applications. Therefore, there has been significant research interest in developing novel formulations with various carrier substances that are compatible with these fungal propagules and can enhance the shelf-life and overall efficacy of the *Trichoderma.* To this end, herein, we investigate the use of a variety of biopolymers and nanoparticles for the stabilization of *Trichoderma atrobrunneum* T720 conidia for biological control. The best-performing agents—agar and cellulose nanocrystals (CNC)—were then used in the preparation of oil-in-water emulsions to encapsulate conidia of T720. Emulsion properties including oil type, oil:water ratio, and biopolymer/particle concentration were investigated with respect to emulsion stability, droplet size, and viability of T720 conidia over time. Overall, agar-based formulations yielded highly stable emulsions with small droplet sizes, showing no evidence of drastic creaming, or phase separation after 1 month of storage. Moreover, agar-based formulations were able to maintain ~ 100% conidial viability of T720 after 3 months of storage, and over 70% viability after 6 months. We anticipate that the results demonstrated herein will lead to a new generation of significantly improved formulations for practical biological control applications.

**Graphical abstract:**

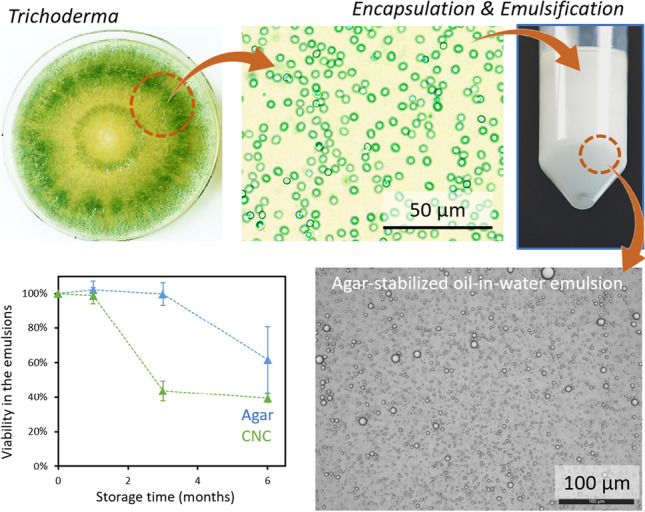

**Key points:**

• *Various biopolymers were evaluated for improving the stability of Trichoderma conidia*

• *Oil in water emulsions was prepared using cellulose nanocrystals and agar as interface stabilizers*

• *Agar-based emulsions showed ~ 100% viability for encapsulated conidia after 3 months of storage*

**Supplementary Information:**

The online version contains supplementary material available at 10.1007/s00253-023-12381-y.

## Introduction

Biological control is the practice of using living organisms to encourage healthy plant growth, predominantly by preventing pests such as insects, weeds, parasites, and pathogenic fungi from growing (Stiling and Cornelissen 2005). This practice has become increasingly common over the past few years, due in part to health concerns and governmental regulations over the use of traditional chemical/synthetic pest control agents (Symondson et al. 2002; Fravel 2005; Wang et al. 2015). One of the most thoroughly investigated microorganisms for biological control is *Trichoderma* spp., which are ubiquitous mesophilic fungi characterized by an outstanding ability to colonize a wide variety of environments and out-compete pathogenic fungi (Altomare et al. 1999; Kredics et al. 2003; Sharma et al. 2012). Moreover, *Trichoderma* shows rapid vegetative growth (Kredics et al. 2004) and produces asexual spores (conidia) and thick-walled resting spores (chlamydospores) in large quantities (Lewis and Papavizas 1984). As a result, *Trichoderma* spp. have gained considerable commercial interest as biological control agents for several common plant diseases (Sharma et al. 2009; Elshahawy, and El-Mohamedy 2019) as well as for the prevention of wood decay (Schubert et al. 2008; Schwarze et al. 2012; Ribera et al. 2017). Typically, the *Trichoderma* conidia are used in biological control formulations and should remain non-germinated prior to application in order to maximize efficacy (Wang et al. 2021). Despite the many benefits of *Trichoderma* spp. as a biological control agent, conidia germinate rapidly in aqueous conditions (Faria et al. 2017), necessitating the use of frequent preparation/application cycles, which is often cost-prohibitive for practical industrial use.

Therefore, recent research has focused on the development of formulations or carrier substances that can protect the conidia of *Trichoderma* spp. against desiccation/damage in order to improve stability and decrease germination prior to application (Teixidó et al. 2022). In addition to being economically viable, suitable formulations/carrier substances must yield high survival rates over time, maintain dormancy/viability during prolonged storage (ideally for a minimum of 6 months (Teixidó et al. 2022)), and must not be harmful to the cells themselves. To this end, several types of formulations have been developed and commercialized for *Trichoderma* spp., with varying degrees of success (Woo et al. 2014). Specifically, formulations acting to encapsulate the biological control agent are of particular interest. One of the most widely investigated materials for encapsulation is alginate, due to its facile ionic crosslinking enabling the encapsulation of various materials (Szekalska et al. 2016). Several studies have demonstrated the use of alginate beads leading to improved shelf-life/viability, UV stability, and the preservation of enzymatic activity (Mancera-López et al. 2019; Maruyama et al. 2020); however, the integration of beads within traditional agricultural irrigation systems is difficult if not impossible, and therefore limits the practical applicability of such formulations.

Another common strategy is the use of various oils as a carrier substances (Peng and Xia 2011; Swarnakumari et al. 2020). In general, oil-based formulations are simple to implement, and importantly are able to subvert the issue of premature conidial germination in aqueous conditions (Luz and Batagin 2005). Moreover, due to the presence of proteinaceous hydrophobins, the chemical nature of most conidia is hydrophobic (Linder et al. 2005), allowing for their natural uptake and encapsulation within an oil phase (Yaakov et al. 2018). However, the use of oils versus aqueous formulations has increased costs and detrimental environmental impacts. Here again, the integration of predominantly oil-based formulations within traditional agricultural irrigation systems is not practical, and therefore severely hinders the applicability of such formulations for industrial use. Therefore, a more recent approach has been the formulation of oil-in-water emulsions, whereby the biological control agent is contained within small oil droplets (typically formed with the help of an emulsifier), the concentration of which is drastically diluted by a surrounding aqueous phase (Xavier-Santos et al. 2011; Yaakov et al. 2018; Herrera et al. 2020). This helps to both bring the overall cost and environmental impact of the prepared formulation down substantially, as water acts as the main carrier substance. Emulsions represent a versatile platform to stabilize/encapsulate various materials, and to date have been investigated for use in applications spanning drug delivery (Tai et al. 2020), food additives (Berton-Carabin and Schroën 2015), cosmetics (Wei et al. 2020), pharmaceuticals (Albert et al. 2019), and cell encapsulation (van Wijk et al. 2014). Some previous studies have also investigated the use of emulsions to encapsulate various microorganisms for biological control applications with success (Bashir et al. 2016; Yaakov et al. 2018).

To this end, herein, we systematically select and evaluate various polysaccharide-based biopolymers and particles as emulsifying agents to encapsulate and stabilize *Trichoderma atrobrunneum* T720 conidia towards biological control applications. In particular, both agar- and (to a lesser extent) cellulose nanocrystal (CNC)-based formulations demonstrated effective stabilizing performance and prolonged the viability of non-germinated conidia for up to 6 months of storage under ambient conditions. Both optimized formulations were able to easily pass through 115 μm filters, which are typically found in industrial irrigation systems, without any noticeable clogging or pressure build-up. Taken together, we anticipate that systematic biopolymer/particle selection and subsequent emulsification represent a promising and relatively straightforward method of encapsulating viable T720 conidia for biological control without causing increased germination or drastically reducing activity.

## Materials and methods

### Preparation of T720 conidial suspensions

T720 conidia were provided by MycoSolutions AG (St. Gallen, Switzerland). Note that the DNA sequence of *Trichoderma atrobrunneum* 720 used in this work has been deposited in GenBankR (Nucleotide LN881560.1) and in the Empa culture collection (*Trichoderma atrobrunneum* T720). Petri dishes (Ø 90mm) containing 17 mL of 4% maltose extract agar (MEA, Oxoid Limited; Basingstoke, UK) were inoculated with the T720 conidia and incubated in a climate chamber at 25 °C and 70% relative humidity (RH) for 14 days. After incubation, plates with visual homogeneous growth were used to prepare spore suspensions. 3 mL of sterile H_2_O containing 0.01% Tween80 (Merk; Buchs, Switzerland) was added to the mature culture plates along with 4 sterile glass beads (Ø 4mm), and the plates were then mixed gently. The conidial suspensions were recovered and filtered through a sterile glass funnel with glass wool into a sterile 50 mL falcon tube. The final concentration of the stock spore suspensions was determined using a hemocytometer. The spore suspensions were stored at 4 °C in the dark prior to use.

### Preparation of biopolymer/particle suspensions

#### Agar

A 250 mL suspension of 0.2% (w/v) agar (ROTH AG; Arlesheim, Switzerland, CAS 9002-18-0) in distilled H_2_O was prepared and stirred for 3 h at room temperature and 600 rpm using a magnetic stir bar. The suspension was then autoclaved for 20 min at 120 °C and stored at 4 °C until use.

#### Carboxymethylcellulose (CMC)

A 250 mL suspension of 0.8% (w/v) ultra-high viscosity, purified grade CMC (Merk; Buchs, Switzerland) in distilled H_2_O was prepared and stirred for 7 h at room temperature and 600 rpm using a magnetic stir bar. The suspension was then sonicated (Branson Ultrasonics S450 Digital Sonifier; Brookfield, CT, USA) at 80% amplitude with 10 s pulses for 2 min 30 s to remove any remaining agglomerates. The suspension was then autoclaved for 20 min at 120 °C and stored at 4 °C until use.

#### Cellulose nanofibers (CNF)

A 3% (w/v) stock CNF suspension (Weidmann Electrical Technology AG; Rapperswil, Switzerland) was diluted to 1.5% (w/v) in distilled H_2_O and stirred for 1 h at room temperature and 600 rpm using a magnetic stir bar. The suspension was then autoclaved for 20 min at 120 °C and stored at 4 °C until use.

#### Cellulose nanocrystals (CNC)

CNC powder (CelluForce; Quebec, Canada) was suspended at 5% (w/v) in distilled H_2_O and homogenized using an IKA T25 ULTRA-TURRAX high shear mixer (Staufen, Germany) operating at 7000 rpm for 1 h. The coarse suspension was then further diluted to 3% (w/v) in distilled H_2_O and stirred for 1 h at room temperature and 600 rpm using a magnetic stir bar. The suspension was subsequently sonicated as above to remove any remaining agglomerates. The suspension was then autoclaved for 20 min at 120 °C and stored at 4 °C until use.

#### Pectin

A 250 mL suspension of 2% (w/v) pectin from the citrus peel in galacturonic acid ≥ 74.0 % (Merk; Buchs, Switzerland) was prepared in distilled H_2_O and stirred for 5 h at 50 °C and 600 rpm using a magnetic stir bar. Due to noticeable degradation of the pectin upon autoclaving, here, only the distilled H_2_O was autoclaved. The final pectin suspension was stored at 4 °C until use.

#### Xanthan

A 250 mL suspension of 0.08% (w/v) xanthan from *Xanthomonas campestris* (Merk; Buchs, Switzerland) was prepared in distilled H_2_O and stirred for 5 h at room temperature and 600 rpm using a magnetic stir bar. The suspension was then sonicated as above to remove any remaining agglomerates, and subsequently autoclaved for 20 min at 120 °C and stored at 4 °C until use.

### Mixing of T720 conidia with the biopolymer/particle suspensions

T720 spore stock solutions (~ 10^8^ spores/mL) were diluted into each of the biopolymer suspensions at a 1:100 ratio to yield ~ 10^6^ spores/mL. The suspensions were then gently mixed for 3 min at room temperature using a magnetic stirrer at 500 rpm. The samples were stored at room temperature (23 ± 2 °C) in the dark until further characterization/use, as outlined below.

### T720 conidial viability in the biopolymer/particle suspensions

Conidial viability (% germination) was characterized over time, up to a 6-month storage period, via a previously published procedure (Paul et al. 1993) with some modifications. Briefly, to trigger germination of viable conidia, samples were incubated in a nutrient media consisting of 30 g/L α-D-glucose (Merk; Buchs, Switzerland), 10 g/L yeast extract (Oxoid Limited; Basingstoke, UK) containing 10 to 12.5% (w/w) total nitrogen derived from amino nitrogen 5.1% (w/w), and 3 g/L NaCl. The media was autoclaved for 20 min at 120 °C, having a final pH of 6.7. Subsequently, 500 μL of each biopolymer-conidial suspension was mixed with 9.5 mL of media in a 100 mL Erlenmeyer flask. The flasks were then incubated (Infors HT AJ125TC Floor Incubator Shaker; Basel, Switzerland) for 16 h at 28 °C and 190 rpm. Afterwards, 40 μL aliquots were pipetted onto a glass slide and observed under optical microscopy (Leica DM400 B LED microscope equipped with a Phase Contrast Condenser and LAS X software (Leica Microsystems, Wetzlar, Germany), under ×10 magnification). Several images were taken per sample in order to accumulate a total spore count of at least 200, counted using ImageJ (Schneider, et al. 2012). Spores were classified as “germinated” if the germ tube was double size of the diameter of the conidia (Hjeljord and Tronsmo 2003). Conidial viability (%) is given by the germination rate (the number of germinated conidia divided by the total number of conidia counted).

### T720 mycelial growth rates

Vegetative growth of T720 in the different biopolymer suspensions was measured every 30 days for up to 6 months of storage. Petri dishes containing 13 mL of 3% (w/v) MEA were spot-inoculated with 20 μL of biopolymer mixture containing approximately 10^4^ colony-forming units (CFU) of T720. The dishes were incubated in the dark in growth chambers at 25 °C and 70% RH for 24 h (Goettel and Inglish 1997). The mycelial growth rates (mm/day) were calculated by measuring the diameter of the inoculated colony after 24 h. A minimum of three technical replicates were used for each biopolymer/time point.

### Concentration of T720 conidia in the biopolymer/particle suspensions

It has been reported that some environmental factors including pH fluctuations or the presence of volatile compounds can trigger *Trichoderma* spp. (micro) conidiation (Steyaert et al. 2010). This becomes particularly prominent if the spores are submitted to conditions associated with elevated stress (Jung et al. 2014). Therefore, to determine the impact of the different biopolymers on the conidiation cycles and germination of the T720, the concentration of conidia (expressed as number of conidia per mL of biopolymer) was recorded monthly for up to 6 months of storage. For this test, a dilution of 1:5 in the water of each formulation was performed and the concentration of conidia was determined manually using a hemocytometer. Per recent analysis, typical error for this type of measurement is in the range of 10% (Manzini et al. 2022) and has been used here.

### Biopolymer water activity (aW)

Water activity for select biopolymer/particle suspensions containing T720 conidia was measured after 6 months, whereby measurements were performed at 25 °C by Microbact AG (Langenthal, Switzerland) following ISO 21807.

### Biopolymer testing in irrigation systems

To determine biopolymer suitability within agriculturally relevant irrigation systems, a model closed-loop system was designed using 4 different ¾” manual disc filters (Netafim Ltd; Kibbutz Hatzerim, Israel) with particle filtration ranges of 115, 130, 200, and 400 microns. A total volume of 20 L of a given biopolymer suspension with T720 was loaded into the system at a dilution of 1:100 from the prepared stock suspensions. Pressure in the system was monitored over time in order to observe any potential clogging of the filters or other issues associated with filtration/pumping of the suspensions.

### Preparation and physical characterization of oil-in-water (o/w) emulsions

As agar and (to a lesser extent) CNC performed the best in previous experiments, these two biopolymers were chosen for the preparation of emulsions. For the aqueous phase, CNCs were used at a concentration of 1%, 2%, or 3% (w/v), while agar was used at a concentration of 0.1%, 0.2%, or 0.3% (w/v). For the oil phase, sunflower, canola, and peanut oil were tested (all purchased at a local supermarket, Migros supermarket; St. Gallen, Switzerland) at oil:water ratios of 30:70, 20:80, and 10:90. Emulsions were prepared in sterile 50 mL falcon tubes by first vortex mixing the formulations for 20 s to form coarse emulsions, and subsequently sonicating each formulation with a Branson S450 Digital Sonifier operating at an amplitude of 25% for 2 min, with 3 s pulses. The formed emulsions were stored in the dark at room temperature until further characterization/use. Emulsions were characterized via optical microscopy (Leica DM400 B LED microscope) to determine emulsion size, and via digital image, capture to analyze stability over time. Here, formed emulsion suspensions were transferred to test tubes, sealed, and stored at room temperature for up to 4 weeks, whereby pictures were taken over time to determine emulsion stability. As above, select emulsion formulations were tested in a model irrigation system with 115-micron filters.

### Imaging and germination of conidia in formulated emulsions

For visualizing conidia in emulsions after 6 months, 0.5 mL of select emulsion formulations were placed with a sterile pipette onto glass slides and sealed with cover slips. The emulsions were analyzed using a Leica DM400M microscope operating in both bright field and dark field modes. Conidial germination was investigated in solid media due to challenges with differentiating and counting conidia within emulsion droplets. Here, 3% (w/v) potato dextrose agar (PDA, Oxoid Limited; Basingstoke, UK) was added to Petri dishes (Ø 90mm), whereby 20 μL per plate of a 1:1000 serial dilution of each emulsion formulation was subsequently added and distributed homogeneously using 4mm Ø sterile glass beads, in order to have a range between 40 and 70 spores per plate. The plates were incubated in the dark for 23 h at 25 °C and 70% RH. The number of germinated conidia was recorded after 1 day of storage (t0), and after 1 month, 3 months, and 6 months. The number of germinated conidia at t0 was used as a baseline to calculate the % viability over time, as above. A minimum of four replicates were tested per sample.

## Results

**Fig. 1:**
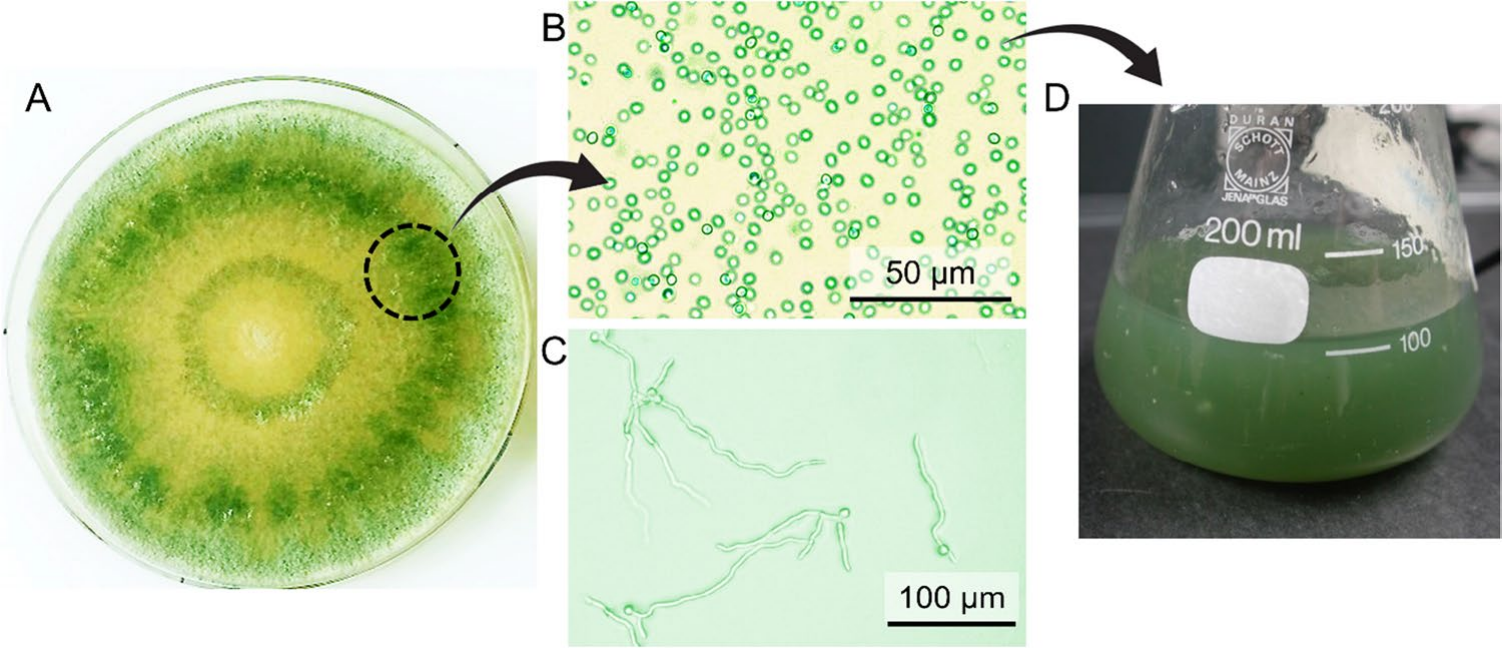
(**A**) Representative image of *T. atrobrunneum* grown in Petri dishes on 3% MEA, microscopy images of (**B**) non-germinated and (**C**) germinated conidia, (**D**) conidial suspensions in water

Suspensions of T720 conidia were cultured, purified, and mixed with various biopolymers in order to evaluate their efficacy as carrier substances/encapsulating agents, as shown schematically in Fig. 1. Preparation of biopolymer suspensions was similar for all tested formulations; however, carboxymethyl cellulose (CMC), cellulose nanocrystals (CNC), and xanthan all required an additional sonication step to remove aggregates, whereas agar, cellulose nanofibers (CNF), and pectin were readily mixed. All biopolymers except for pectin were sterilized effectively via autoclaving, ensuring bacterial contamination could be minimized; pectin appeared to degrade due to the autoclaving conditions and could therefore not be sterilized.

For all biopolymer mixtures, an initial viability screening was performed after 24 h (Table 1). Agar, CMC, CNF, and CNC all showed conidial viability in excess of 90%, indicating no substantial negative effects on the conidia. Representative microscopy images used for this calculation are shown in the Supporting Information, Fig. S1. Note that due to the highly fibrillar/networked structure of the CNF-based samples, a lactophenol blue stain was necessary to distinguish the germinated conidia from CNF for viability calculations. When evaluating samples with both xanthan and pectin, extensive conidial germination and hyphal aggregation were apparent (Supporting Information, Fig. S1), indicating poor stabilizing performance. For this reason, both xanthan and pectin were eliminated as potential candidates in further characterizations.

**Table 1:** Screening of biopolymers with T720 conidia after 24 h. Aggregation (see Supporting Information, Fig. S1) did not allow for proper characterization of pectin or xanthan

Biopolymer	Germinated spores	Non-germinated spores	Viability (%)
Agar	220	4	98.2
CMC	240	18	93.0
CNF	228	6	97.4
CNC	225	13	94.5
Pectin	–	–	–
Xanthan	–	–	–

**Fig. 2:**
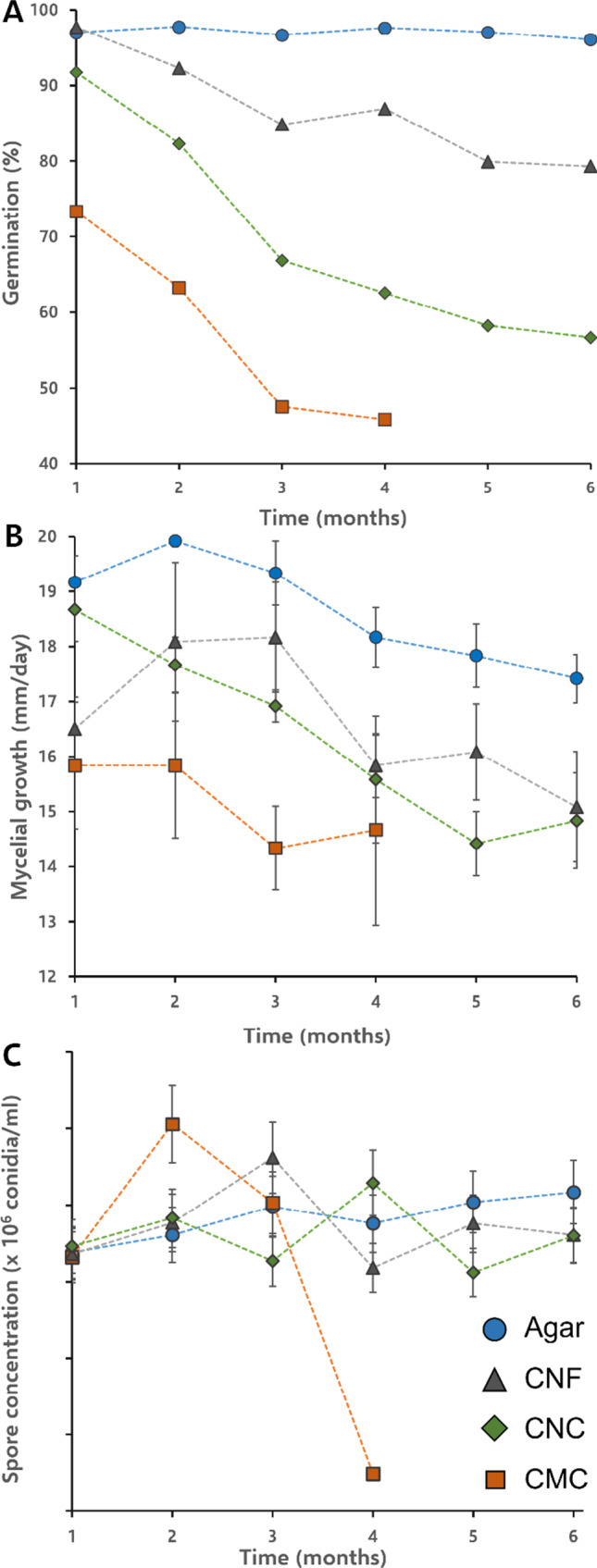
(**A**) Germination of T720 conidia, calculated from manually counting at least 200 spores per sample condition, (**B**) mycelial growth rate, with data represented as the average and error bars representing the standard deviation of 3 measurements, and (**C**) spore concentration over time, counted using a hemocytometer and error bars representing 10% error, per a previous report (Manzini et al. 2022). All lines are guides for the eye

Extended viability screening was performed on agar, CNC, CNF, and CMC suspensions for up to 6 months of room temperature storage (Fig. 2A). These remaining 4 biopolymer mixtures looked relatively homogenous in appearance (Supporting Information, Fig. S1), with little conidial aggregation apparent even after 6 months of storage in Falcon tubes. However, some settling of conidia and mycelial growth was evidenced in the CMC and CNC mixtures. For the cellulosic biopolymer samples (CNF, CNC, and CMC), conidial viability dropped steadily over time to ~ 79%, ~ 57%, and below 45% respectively after 6 months of storage (note that viability values were no longer measured below 45%). This is in stark contrast to agar, which demonstrated a remarkable sustained viability of ~ 96% after 6 months of storage. Mycelial growth rates (Fig. 2B) showed a similar trend, with agar formulations consistently demonstrating the highest growth rates. Note that for all cases (except CMC), conidia concentration remained relatively stable over the 6 months of storage, indicating a healthy balance between conidiogenesis and germination (Fig. 2C) (Steyaert et al. 2010).

The agar, CNC, and CNF formulations were then tested in model irrigation systems to determine their suitability for practical use in industrial biological control applications. Here, a model filtration system was developed with four interchangeable disc filters. Disc filters were chosen, as they are one of the most commonly used filters in agricultural irrigation systems (Feng et al. 2020). All formulations were able to pass through the 400 μm filters without causing any pressure increase in the system, measured for up to 40 min (baseline pressure of ~ 0.65 bar maintained). However, upon decreasing the filter size to 200 μm, a slow pressure increase was observed for the CNF formulation, indicating material build-up on the filter (Supporting Information, Fig. S2). This pressure increase was even more significant when using a 130 μm filter. In both cases, this build-up was noticeable by the eye when the filters were removed and examined (Supporting Information, Fig. S2). This indicates that CNF-based formulations would be challenging to use in existing industrial irrigation equipment. This is also an indication that CNF-based formulations may also not be suitable for foliar treatments, where backpack sprayers are used and are highly sensitive to clogging (Sesan et al. 2020; Szwedziak et al. 2020). Conversely, both the CNC and agar-based formulations did not show any signs of clogging/build-up, even down to the smallest filter size tested (115 μm), indicating that these formulations could have applicability for a variety of practical biological control scenarios.

**Fig. 3:**
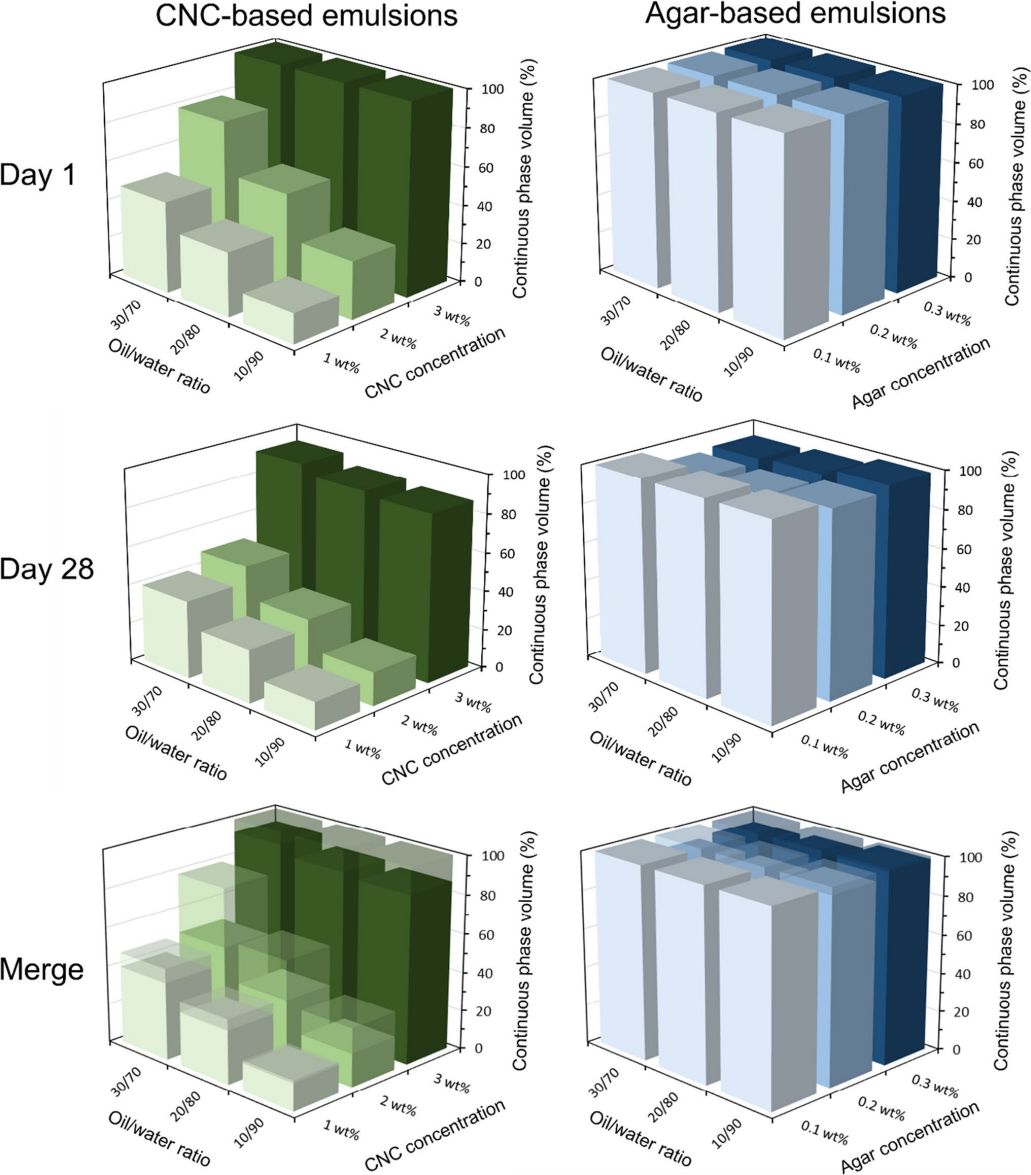
Emulsion continuous phase volume (%) for CNC- and agar-stabilized canola oil-in-water emulsions. The effect of oil:water ratio and biopolymer concentration (wt% in water phase) are investigated 1 day and 28 days post-preparation. A merged graph is also shown to demonstrate the change in emulsion continuous phase volume attributed to creaming over the duration of the experiment (over 28 days). All data can also be viewed as scatter plots in the Supporting Information, Fig. S5

As agar and CNC both performed the best in previous experiments, they were chosen as the preferred candidates to form emulsions. Here, emulsion formulation properties including oil type, oil:water ratio, and biopolymer concentration were varied in order to determine their effects on emulsion stability. Emulsion stability was analyzed by measuring the height of the continuous (intact) emulsion phase 1 and 28 days post-preparation (Fig. 3, Supporting Information Figs. S3 – S5). For CNC stabilized emulsions, increasing the oil:water ratio from 10:90 to 30:70 resulted in an increase in the continuous phase volume (decrease in creaming) across the 28-day observation period, indicating increased emulsion stability. This is consistent for several oil-in-water emulsions, whereby increasing the water content leads to increased creaming and thus decreased emulsion stability (Goodarzi and Zendehboudi 2019). No large differences were apparent upon changing the selection of the type of oil between canola (Fig. 3), sunflower (Supporting Information, Fig. S3), and peanut (Supporting Information, Fig. S4) oils, albeit the use of peanut oil resulted in a systematic decrease in continuous phase volume, attributed to its increased viscosity compared to the other oils, and thus greater propensity for creaming (Diamante and Lan 2014). Finally, increasing the CNC concentration from 1 to 3 wt% resulted in significant increases in the continuous phase volume, likely due to increased network formation of the CNCs increasing suspension viscosity and providing enhanced resistance to creaming (Goodarzi and Zendehboudi 2019; Kedzior et al. 2020). For agar-stabilized emulsions, regardless of agar concentration, oil type, or oil:water ratio, little evidence of creaming was apparent, with the continuous phase remaining exceptionally stable, staying above 90% after 28 days of storage.

**Fig. 4:**
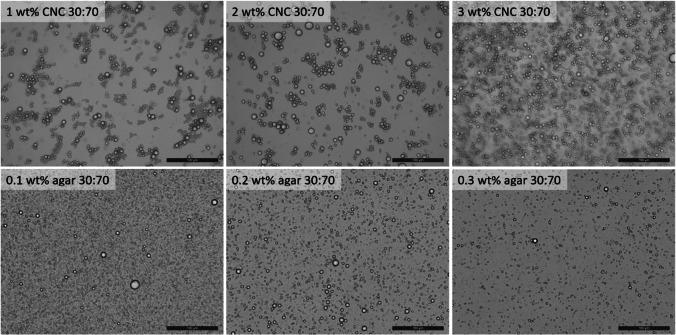
Microscopy images of emulsion droplets for CNC- and agar-stabilized canola oil-in-water emulsions after 28 days of storage. The effect of changing biopolymer concentration is shown. All scale bars are 100 μm

Emulsions were further characterized via microscopy in order to directly observe emulsion size and interactions between droplets (Fig. 4). For both CNC- and agar-stabilized canola oil-in-water emulsions, droplet size remained relatively constant as the biopolymer concentration was increased. However, differences in emulsion morphology were clearly evidenced between the CNC-stabilized emulsions and agar-stabilized emulsions. Agar-stabilized emulsions had a smaller droplet size, with no significant evidence of flocculation, while CNC-stabilized emulsions were larger and tended to clump together. These trends related to emulsion size were also consistent across varying oil:water ratios (Supporting Information, Fig. S8). It should also be noted that minimal changes in emulsion droplet size were observed between Day 1 and Day 28, even though creaming was evidenced (especially for CNC-stabilized emulsions) during this timeframe (Supporting Information, Fig. S9). This also supports creaming as the main mechanism of emulsion instability, as this phenomenon is generally recognized for not leading to changes in particle size (Goodarzi and Zendehboudi 2019; Pal 2019).

**Fig. 5:**
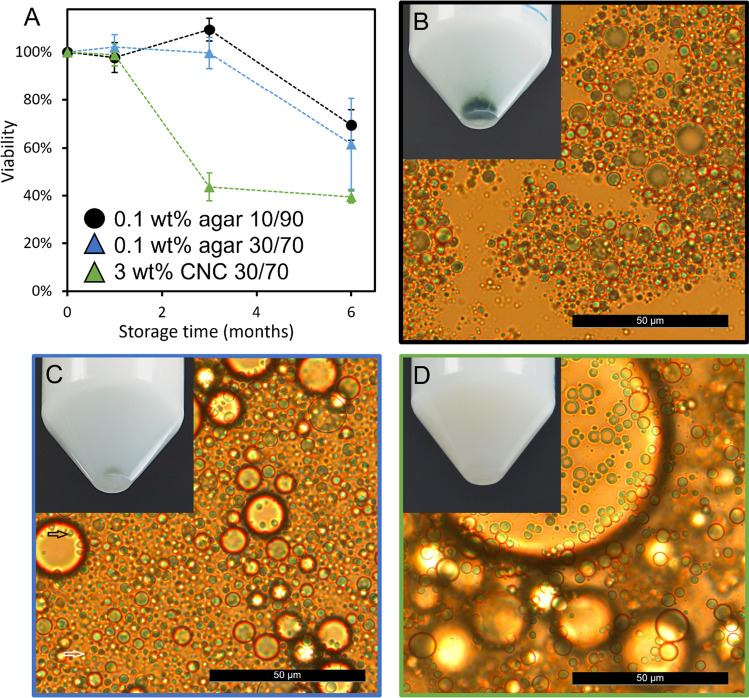
(**A**) Viability of T720 conidia encapsulated within biopolymer emulsions, demonstrating the effect of canola oil:water ratio and biopolymer concentration on T720 germination; results are presented as the average, with error bars representing the standard deviation. B–D Microscopy and optical images after 1 month of storage of (**B**) 0.1 wt% agar 10:90, (**C**) 0.1 wt% agar 30:70, and (**D**) 3.0 wt% CNC 30:70 canola oil-in-water emulsions. Falcon tubes in B-D are 50 mL; all scale bars are 50 μm. Additional microscopy images can be seen in the Supporting Information, Fig. S10

Based on overall stability, three canola oil-in-water emulsion formulations were chosen, whereby T720 conidia were added directly following emulsion formation via simple vortex mixing. This was done following a previously published method in order to minimize stress and potential loss of viability due to probe sonication (Yaakov et al. 2018). Conidia viability was then assessed over time following an established method (Atanasova et al. 2013), for up to 6 months of storage under ambient conditions (Fig. 5A). Here, both agar- and CNC-stabilized emulsion droplets appear larger due to the addition of conidia. However, as seen for empty emulsions, agar-stabilized droplets are again smaller than the CNC-stabilized droplets, indicating increased emulsion stability. The 3.0 wt% CNC-stabilized emulsions (30:70 oil:water ratio) were able to maintain conidia viability for 1 month of storage; after 3 months viability dropped to ~ 40%, and stabilized at this value for the remainder of the experiment (6 months). In contrast, 0.1 wt% agar emulsions (30:70 and 10:90 oil:water ratio) maintained a conidia viability of ~ 100% for up to 3 months of storage. Viability then decreased to ~ 70% after 6 months. Interestingly, noticeable sedimentation of the conidia was evidenced after 1 month in the 10:90 agar-stabilized emulsions (Fig. 5B). We attribute this to the relatively small volume of oil used in this formulation, not allowing for proper encapsulation of the added T720 conidia. Sedimentation was much less evident in the 30:70 agar-stabilized emulsions (Fig. 5C), yet interestingly, this sedimentation had little effect on overall viability. Although out of scope for the current research, this indicates that optimizing the concentration of encapsulated conidia in order to encourage complete uptake into the oil phase and prevent excess sedimentation could improve overall viability and practical use of such formulations in biological control applications.

Notably, both 30:70 canola oil-in-water emulsions stabilized by CNC or agar (without encapsulated *Trichoderma* conidia) were able to pass through our model irrigation system with 115-micron filters, whereby no pressure change was evidenced in the system for up to 40 min. Furthermore, microscopy analysis demonstrated that no significant changes in emulsion size/aggregation/breaking occurred (Supporting Information, Fig. S11), suggesting that this is a viable encapsulation and delivery method for practical biological control applications.

## Discussion

In order to better understand the stabilizing effects of agar, CNC, and CNF on *Trichoderma* conidia, the water activity for each biopolymer suspension was determined after 6 months of storage. Water activity gives a measure of the relative amount of water available for biochemical reactions, with a high water activity (1 = pure water) generally associated with increased metabolic activity and microbial growth, and therefore decreased product shelf-life. Here, agar showed the lowest water activity (0.972), with CNF and CNC showing higher values (0.985 and 0.986 respectively), supporting the increased stability of the agar formulations versus the others. Note that although a low water activity is desired to maintain the dormancy of the conidia, a water activity of ≤ 0.95 is considered extremely low for such applications, and has been previously shown to hinder microbial growth (Schwarze et al. 2012).

Moreover, *Trichoderma* is well-known to express cellulolytic enzymes (cellulases) which break down cellulosic material (Zhou et al. 2008; Shafique et al. 2009; Colussi et al. 2011). The use of CNC, CNF, or CMC (cellulose derivatives) as carrier substances could therefore trigger the secretion of cellulases and the spontaneous germination of conidia. Once germination is triggered, the accumulation of toxic metabolism-derived products could result in the eventual decrease of viable spores as observed herein. As CMC is a suspension of individualized cellulose polymer chains, while CNC and CNF are both nanoparticles comprised of cellulose chain bundles, likely the *Trichoderma* cellulases can more readily break down the CMC cellulose chains, resulting in its comparatively much poorer performance. Importantly, although several microorganisms are known to express agarases (Fu and Kim 2010; Wenjun et al. 2016; Khalifa and Aldayel 2019; Fawzy et al. 2020), this has not been reported for *Trichoderma* spp., which may also help explain the superior stability/viability of conidia in the agar-based formulations as compared to the cellulose-based formulations.

Notably, as an emerging sustainable nanomaterial (De France et al. 2017, 2021), CNCs have been extensively investigated as a high-performance emulsifier (Kedzior et al. 2020); however, the use of agar for such applications has been significantly less explored. Emulsion instability, observed as a decrease in the overall continuous phase volume due to creaming, sedimentation, oil leakage, or phase separation, leads to a drastic decrease in material performance (Kedzior et al. 2020); thus emulsions with the largest overall continuous phase are desired. Here, creaming was identified as the main cause of emulsion instability (McClements 2007; Pal 2019), as the less-dense dispersed oil phase can be seen to slowly migrate and concentrate towards the top of the tube over time (representative optical images for CNC- and agar-stabilized emulsions in canola oil 1 and 28 days post-preparation can be seen in the Supporting Information, Figs. S6 and S7). This phenomenon was much more evident for CNC-based emulsions than for agar-based emulsions, indicating that agar is a more suitable interface stabilizer. Moreover, from microscopy analysis, the CNC-stabilized emulsion droplets had a larger size and tended to flocculate together, which could also contribute to their decreased stability versus the agar-stabilized emulsions (Goodarzi and Zendehboudi 2019). In general emulsion droplet size is a good indicator of emulsion stability, with smaller droplets typically indicating a more stable emulsion (Kedzior et al. 2020). This improved stability of the agar-based emulsions, along with the general compatibility of *Trichoderma* conidia with the agar itself, is likely the primary reason for the significantly enhanced viability observed in the agar-stabilized emulsions.

In conclusion, various biopolymers were investigated for the stabilization of *T. atrobrunneum* T720 conidia towards biological control applications. Overall, agar and cellulose-based formulations (CNC, CNF, CMC) showed promise for stabilizing T720 conidia, with viability values in excess of 90% and minimal aggregation of conidia after 24 h of storage under ambient conditions. After 6 months of storage, conidial viability remained high for agar-based formulations (> 95%) but dropped significantly for CNF (~ 80%), CNC (~ 60%), and CMC (0%). Due to the entangled fiber-like networked structure of CNF, these formulations led to significant clogging in model irrigation systems, while both CNC and agar-based formulations were readily filtered with no significant increase in pressure within the irrigation system. As a result, oil-in-water emulsions were prepared with both CNC and agar, whereby agar-based emulsions at varying agar concentrations and oil:water ratios remained stable for up to 1 month of storage, without any significant evidence of emulsion creaming or phase separation (~ 100% continuous phase). For CNC-based emulsions, higher CNC concentrations and higher oil:water ratios led to improved emulsion stability, with 3 wt% CNC, 30:70 canola oil-in-water emulsions showing > 90% continuous phase after 1 month of storage. Droplet size was smaller for agar-stabilized emulsions, with no evidence of droplet flocculation, in contrast to CNC-stabilized emulsions. Viability of T720 conidia in the CNC-based emulsions was ~ 100% after 1 month of storage at ambient conditions, dropping to 40% after 3 months. In contrast, conidia viability in the agar-based emulsions remained at 100% for 3 months of storage, finally dropping to ~ 70% after 6 months.

## Supplementary information

ESM 1

## Data Availability

Data will be made available upon reasonable request to the authors.
