# Supplementary material for: Biopolymer-based emulsions for the stabilization of Trichoderma atrobrunneum conidia for biological control
Source: Appl Microbiol Biotechnol. 2023 Jan 23;107(4):1465–76. doi: 10.1007/s00253-023-12381-y (PMC9898383; doi:10.1007/s00253-023-12381-y)
Supplement: Supplementary file 1 — ESM 1 [file 253_2023_12381_MOESM1_ESM.pdf]

Supporting Information

Biopolymer-Based Emulsions for the Stabilization of *Trichoderma atrobrunneum* Conidia for Biological Control

Yolanda Martínez,[1] *Markus Heeb,*[1] *Tine Kalač,*[1] *Zennat Gholam,*[1] *Francis W.M.R. Schwarze,*[1*] *Gustav Nyström,*[2,3*] *Kevin De France,*[2,4*]

1. *Laboratory for Cellulose & Wood Materials, Empa – Swiss Federal Laboratories for Materials Science and Technology, Lerchenfeldstrasse 5, 9014 St. Gallen, Switzerland*
2. *Laboratory for Cellulose & Wood Materials, Empa – Swiss Federal Laboratories for Materials Science and Technology, Überlandstrasse 129, 8600 Dübendorf, Switzerland*
3. *Department of Health Science and Technology, ETH Zürich, Schmelzbergstrasse 9, 8092 Zürich, Switzerland*
4. *Department of Chemical Engineering, Queen's University, 19 Division St., Kingston, Ontario K7L 3N6, Canada*

* To whom correspondence should be addressed

E-mail: kevin.defrance@queensu.ca, francis.schwarze@empa.ch, gustav.nystroem@empa.ch

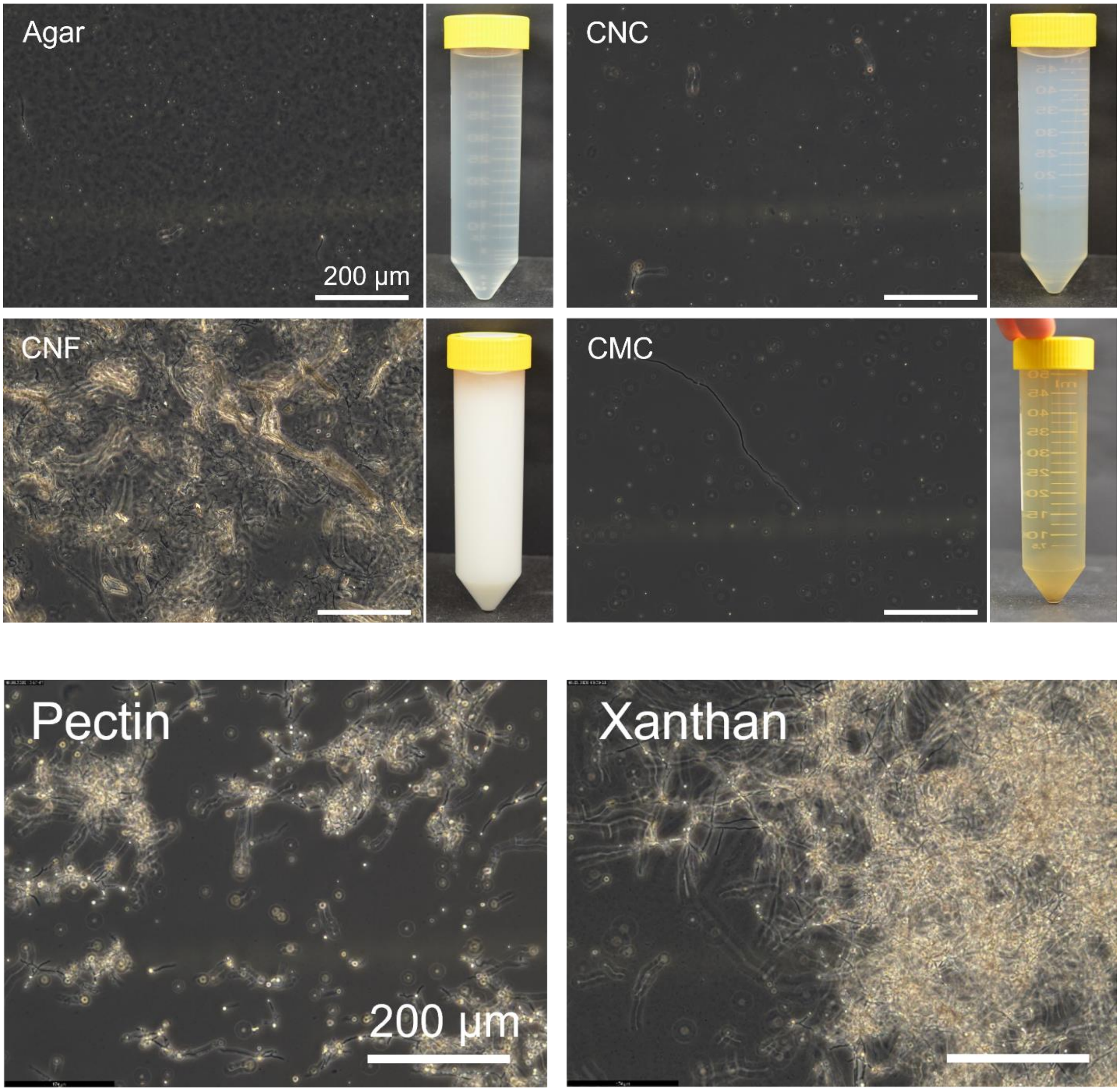

Fig. S1: Representative microscopy images of stock biopolymer suspensions mixed with T720 conidia (~ 10^6 CFU/mL) diluted at a ratio of 1:3 in water after 24 h. Representative optical images of undiluted stock biopolymer suspensions in falcon tubes after 6 months of storage are also shown for select biopolymers. All scale bars are 200 µm.

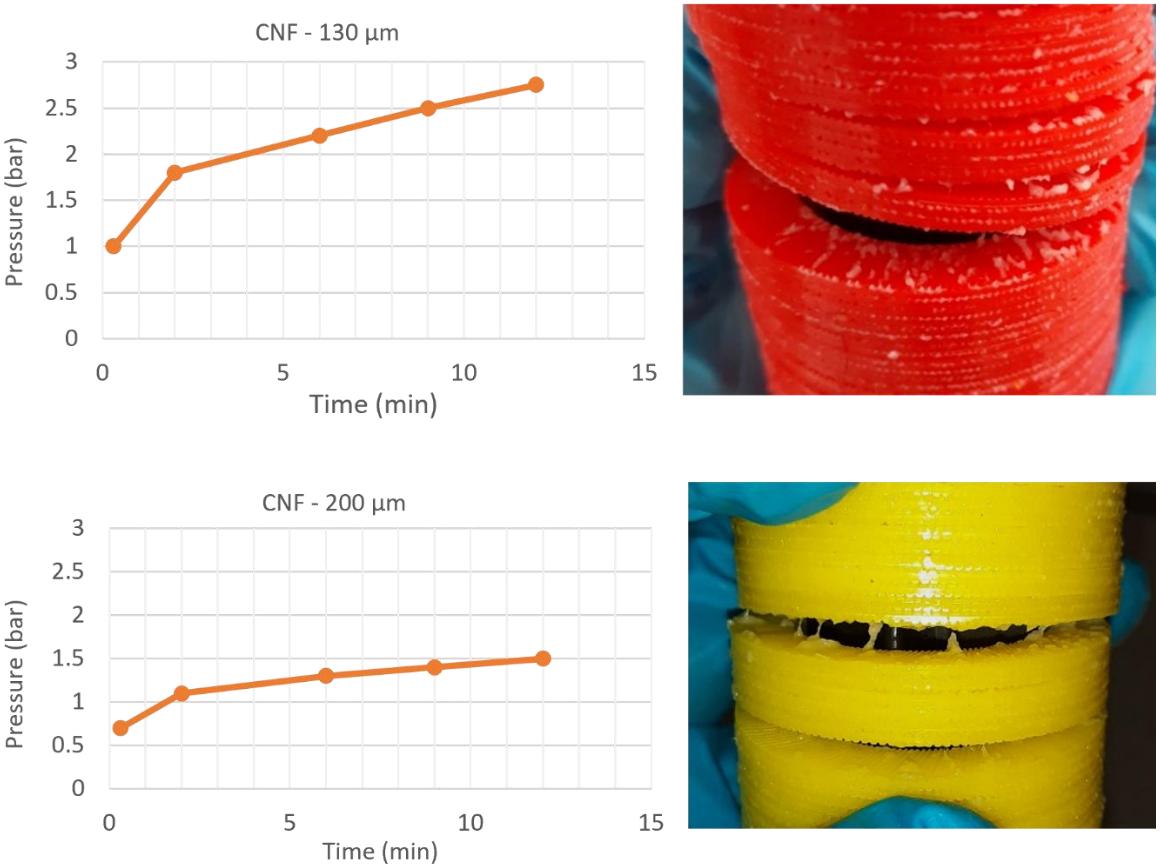

Fig. S2: CNF-based formulations in irrigation systems, showing the pressure change over time for model systems with 130 and 200 µm filters for up to 12 minutes of operation. In both cases, drastic CNF build-up is apparent on the filters after only 12 minutes of operation.

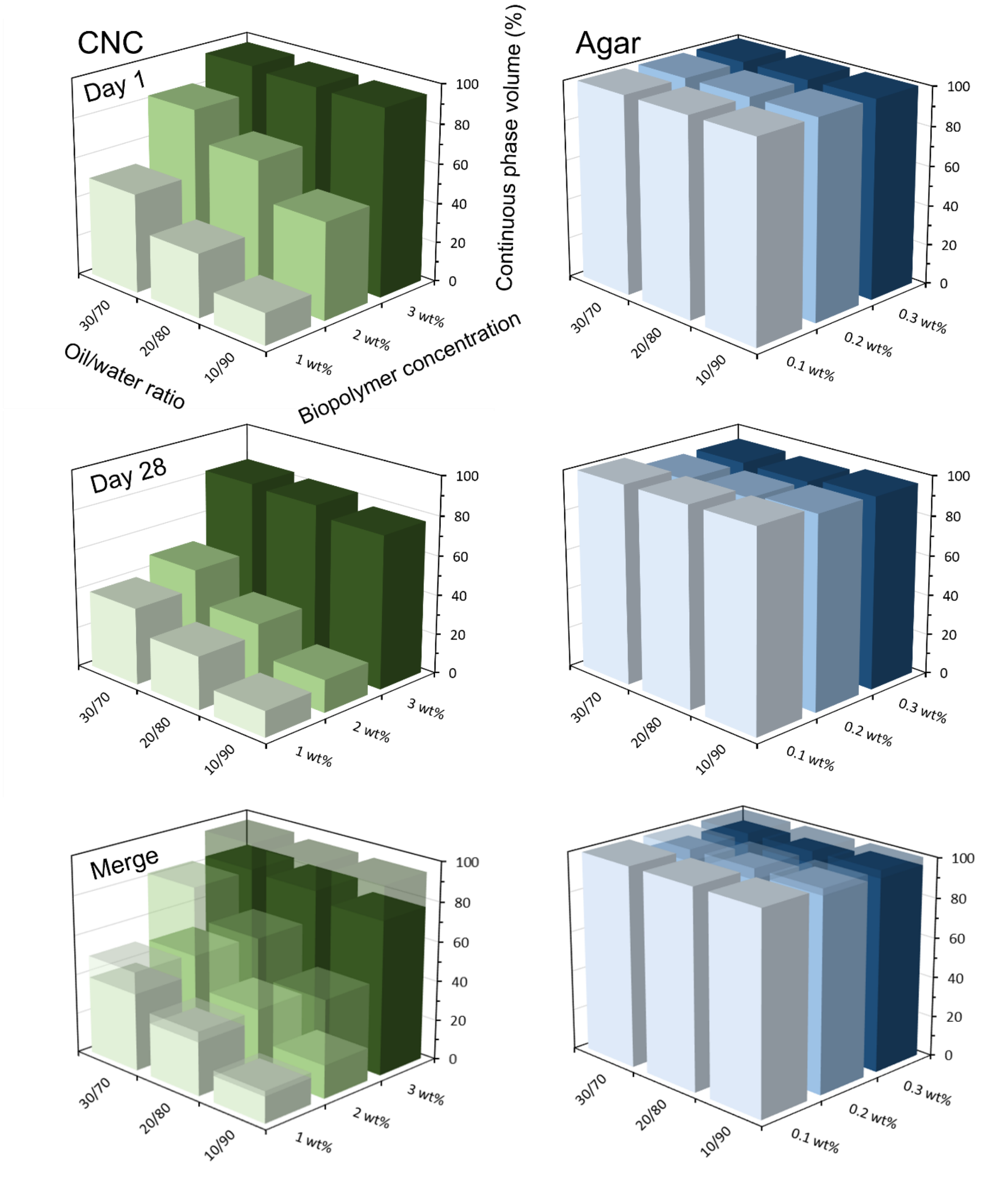

Fig. S3: Emulsion continuous phase volume (%) for CNC- and agar-stabilized sunflower oil-in-water emulsions. The effect of oil/water ratio and biopolymer concentration (wt% in water phase) are investigated 1-day and 28 days post-preparation.

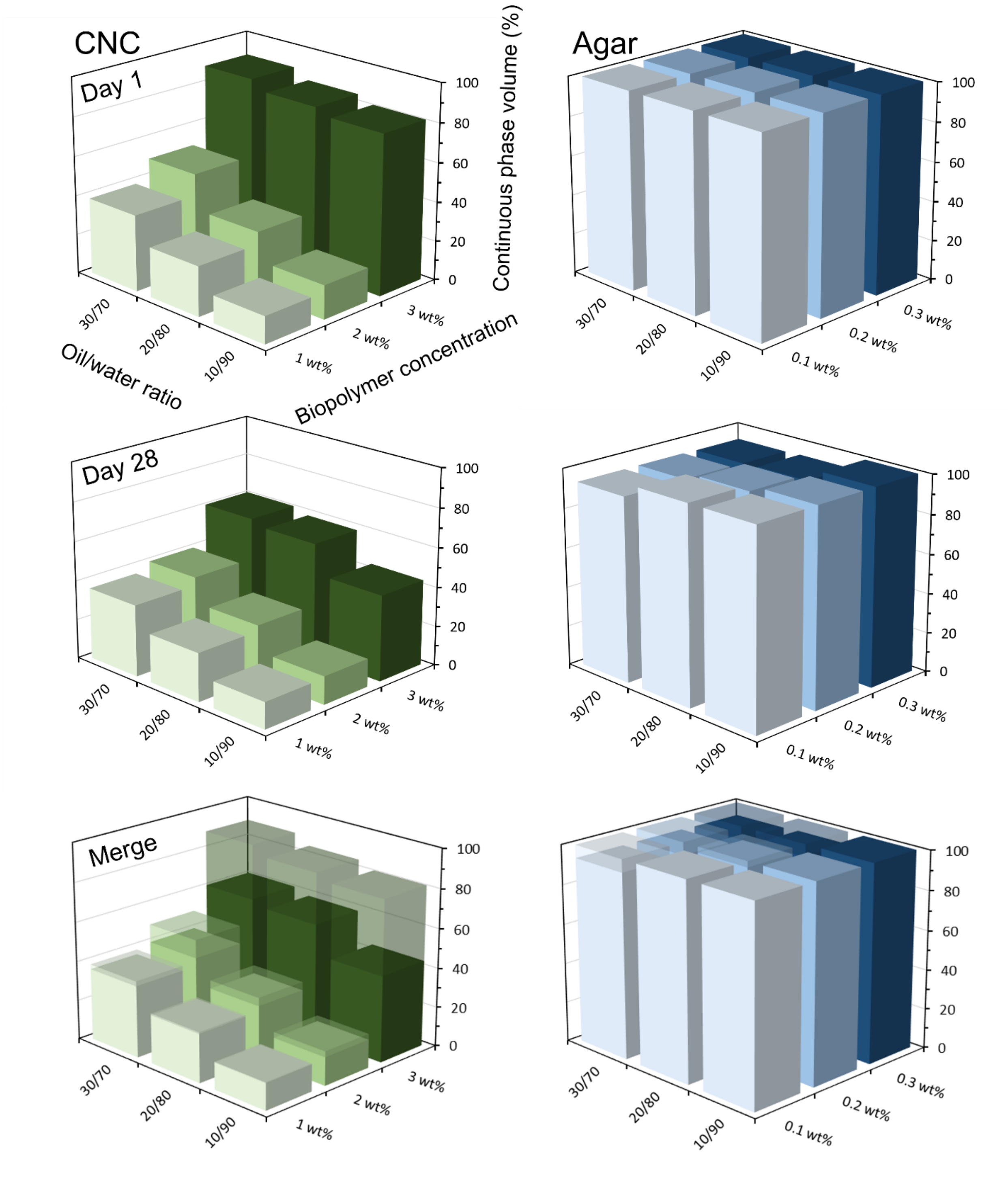

Fig. S4: Emulsion continuous phase volume (%) for CNC- and agar-stabilized peanut oil-in-water emulsions. The effect of oil/water ratio and biopolymer concentration (wt% in water phase) are investigated 1-day and 28 days post-preparation.

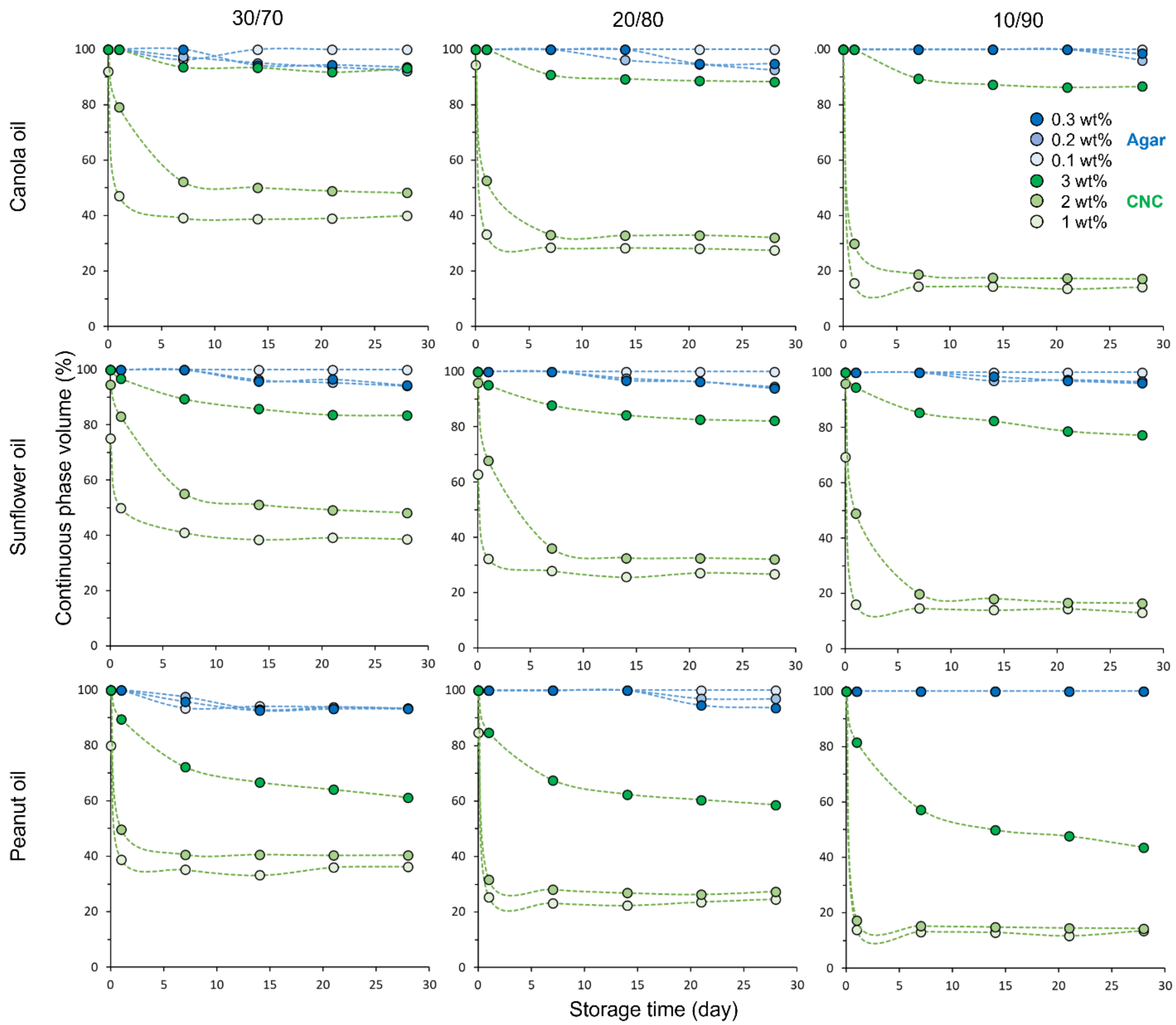

Fig. S5: Evolution of the emulsion continuous phase volume (%) for CNC- and agar-stabilized oil-in-water emulsions over 28 days. The effect of oil type, oil/water ratio, and biopolymer concentration are investigated.

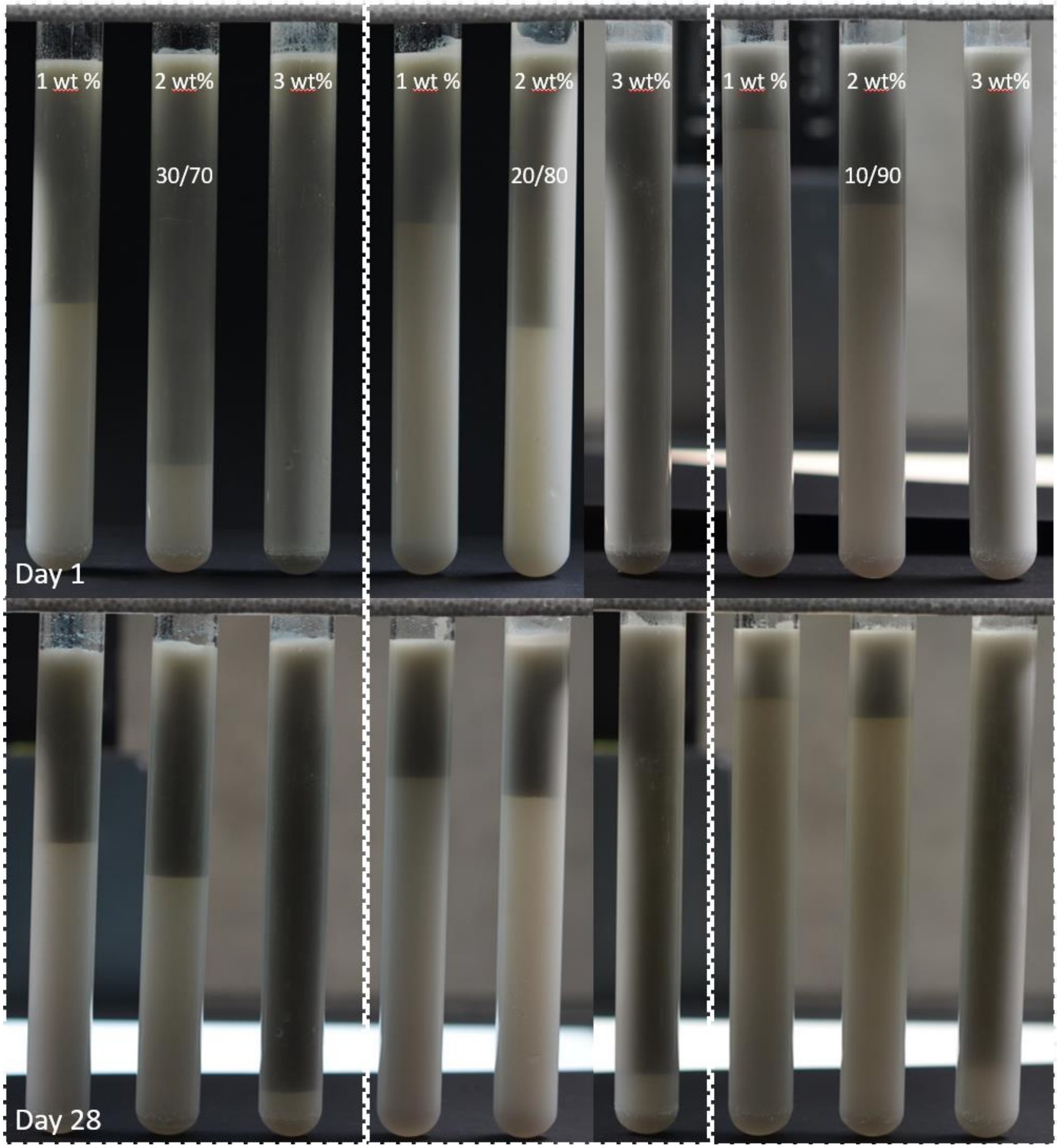

Fig. S6: Representative images of the emulsion continuous phase volume (%) for CNC-stabilized canola oil-in-water emulsions 1 and 28 days post-preparation. The effect of oil/water ratio and CNC concentration are investigated.

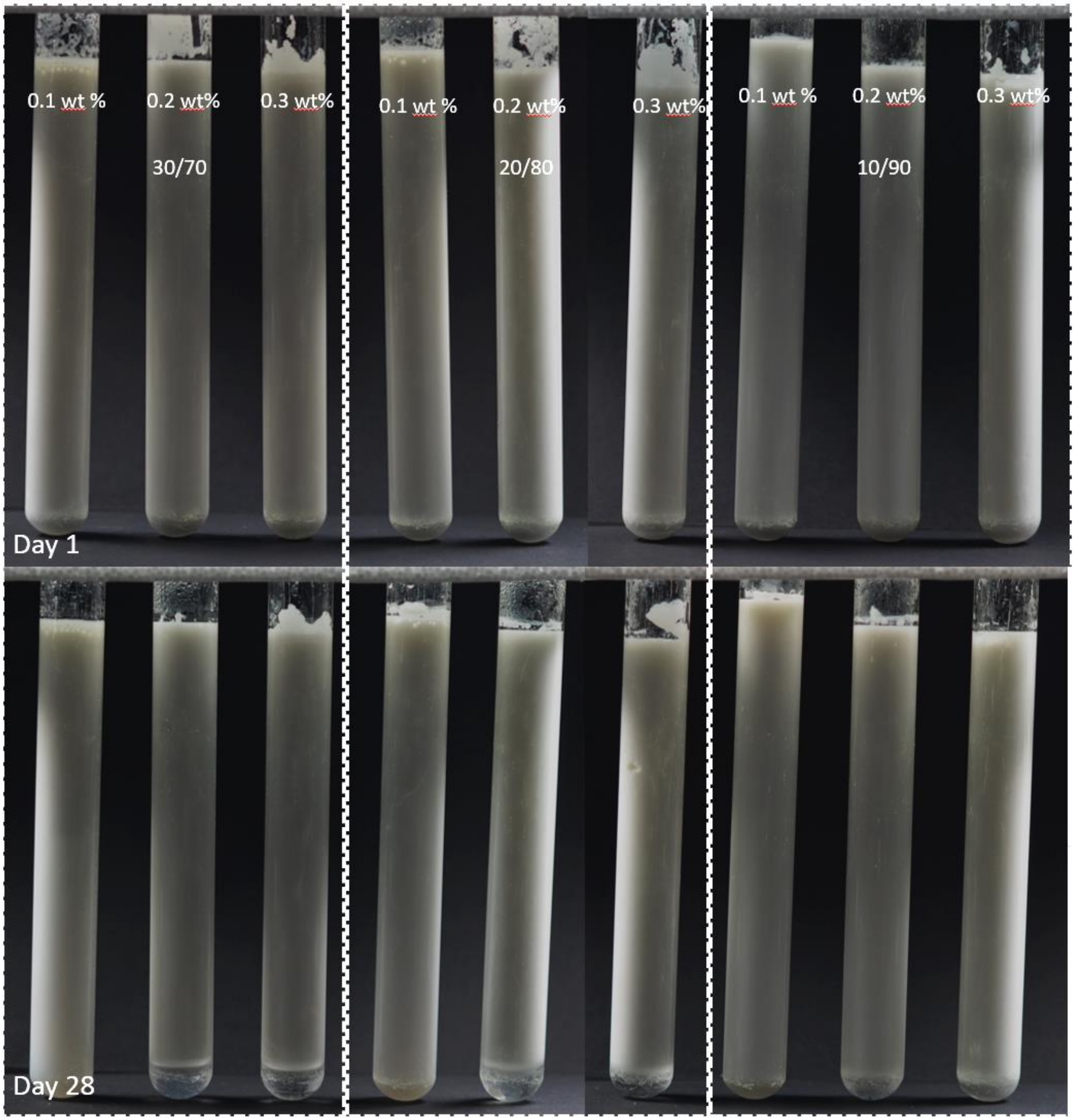

Fig. S7: Representative images of the emulsion continuous phase volume (%) for agar-stabilized canola oil-in-water emulsions 1 and 28 days post-preparation. The effect of oil/water ratio and agar concentration are investigated.

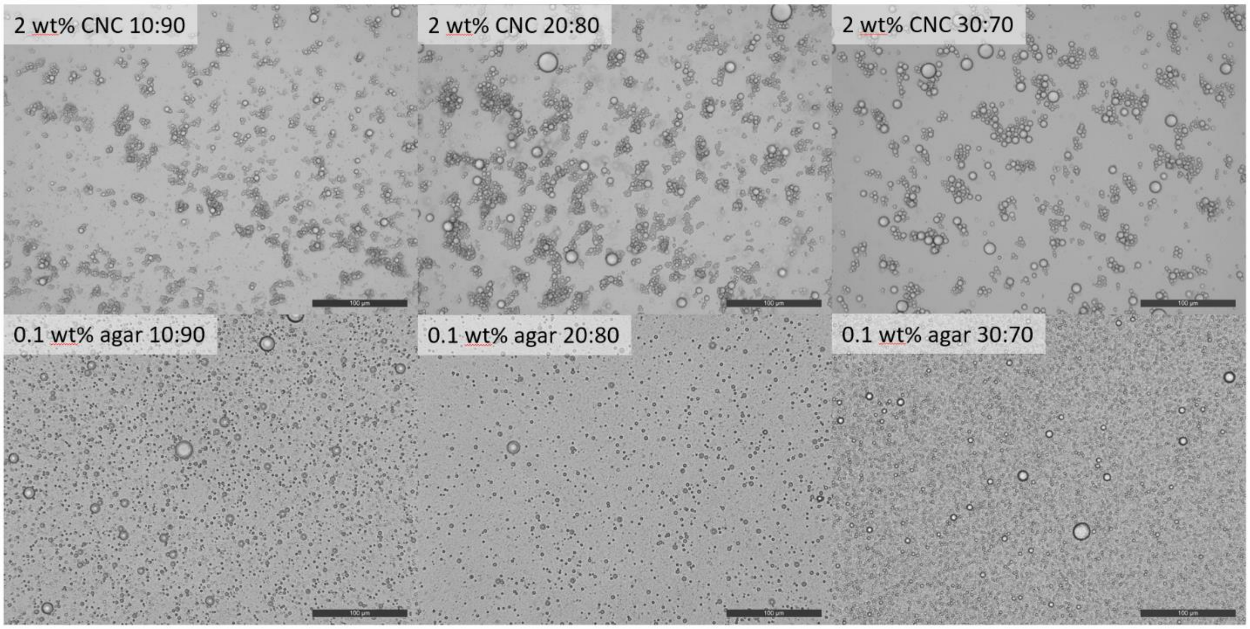

Fig. S8: Microscopy images of emulsion droplets for CNC- and agar-stabilized canola oil-in-water emulsions after 28 days of storage. The effect of changing oil:water ratio is shown. All scale bars are 100 µm.

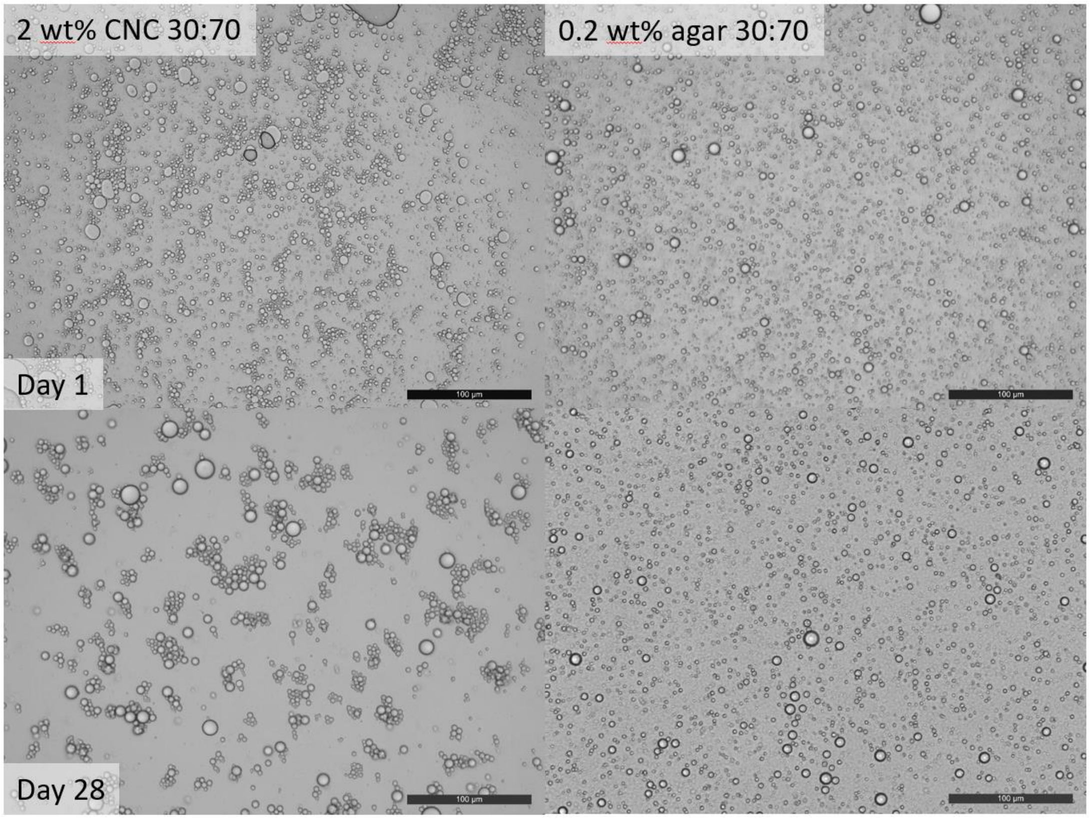

Fig. S9: Microscopy images of emulsion droplets for select CNC- and agar-stabilized canola oil-in-water emulsion formulations after 1 and 28 days of storage. All scale bars are 100 µm.

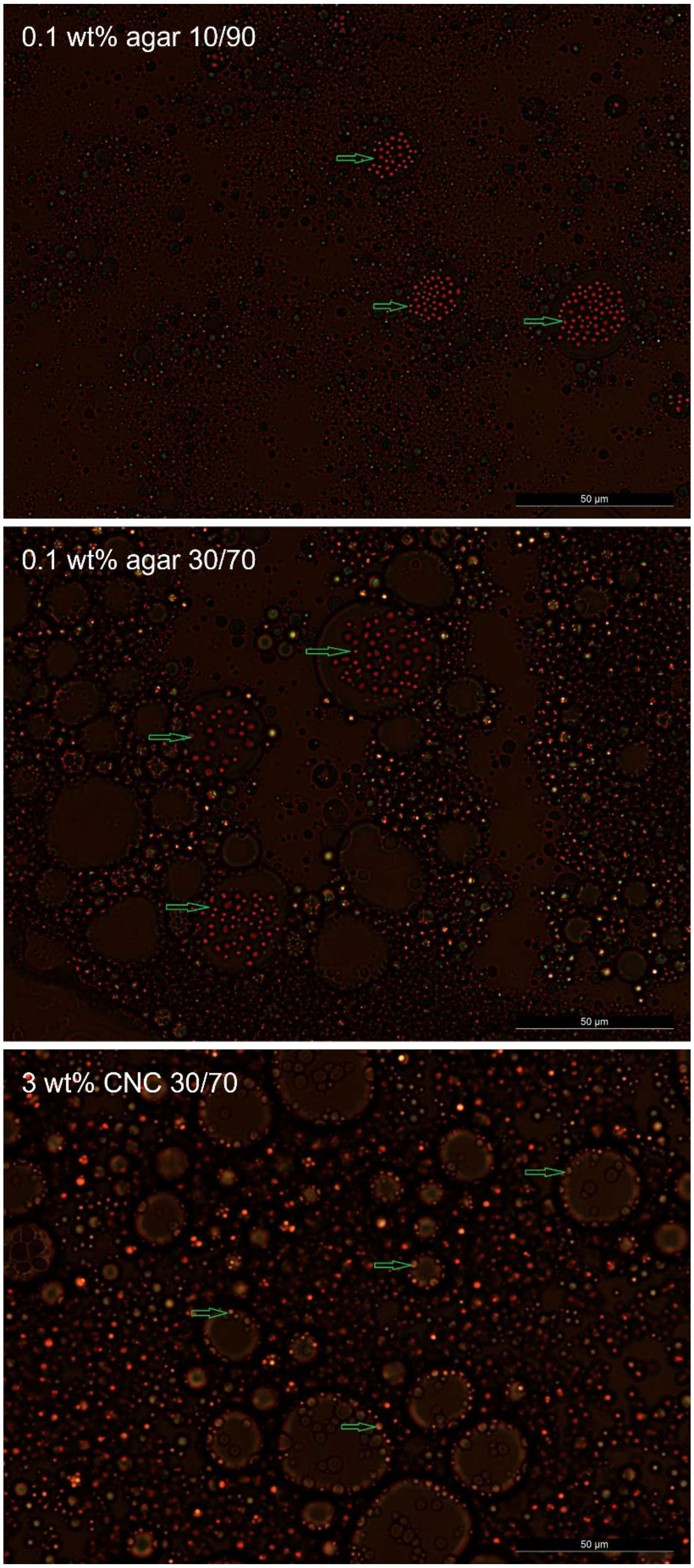

Fig. S10: Dark field microscopy images of T720 conidia encapsulated within canola oil-in-water emulsions after 1 month storage. Several individual conidia are indicated by green arrows. All scale bars are 50 µm.

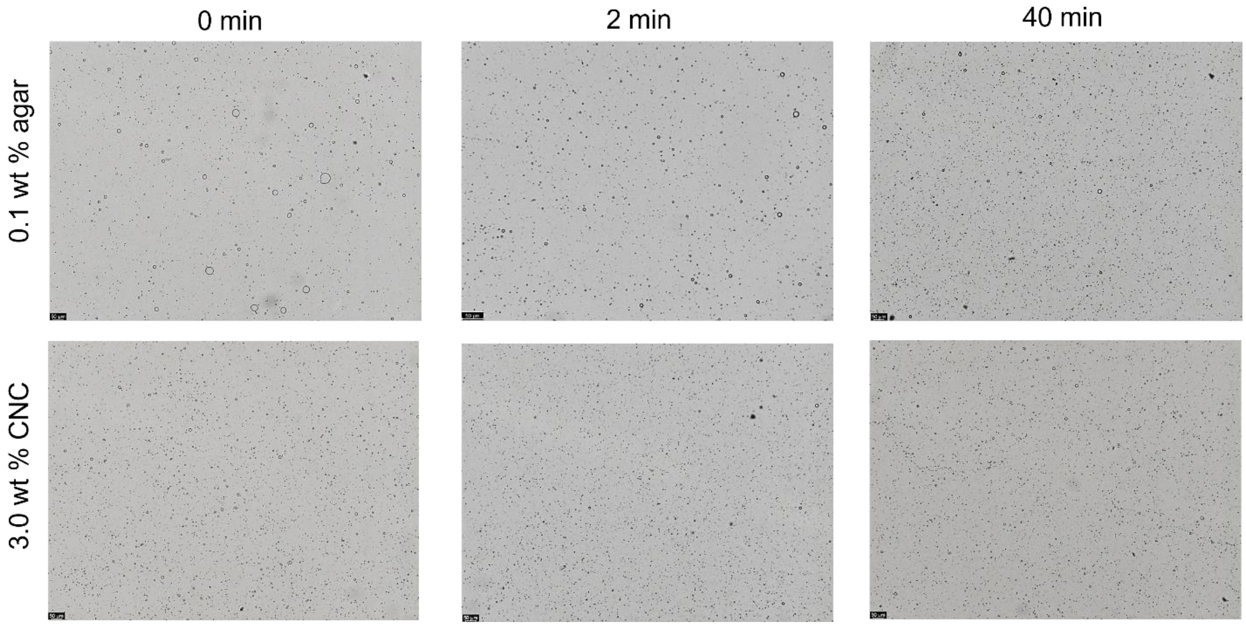

Fig. S11: Representative microscopy images of 30/70 canola oil-in-water emulsions over time in a model irrigation system with 115 µm filters. All scale bars are 50 µm.
